# A descriptive investigation of the impact of statewide distribution policies and consumer vulnerabilities on COVID-19 vaccination in the united States

**DOI:** 10.1007/s10729-025-09727-5

**Published:** 2025-10-04

**Authors:** Kathleen Iacocca, Beth Vallen, Alicia Strandberg, Laura Meinzen-Dick

**Affiliations:** 1 Department of Management & Operations Villanova, Villanova University, Villanova, PA USA; 2 Department of Marketing & Business Law Villanova, Villanova University, Villanova, PA USA; 3 Agricultural, Food, and Resource Economics, Michigan State University, East Lansing, MI USA

**Keywords:** COVID-19, Vaccine distribution, Pandemic preparedness, Pandemic response, Public health policy, Health disparities

## Abstract

This research leverages data from various disparate sources to examine how state-level policy distribution decisions and local, county-level population vulnerability factors likely to hinder vaccination influenced COVID-19 vaccination efforts across the United States. Unlike other nations that coordinated their responses at a national level, this study uses U.S. states and counties as individual units of analysis. This approach allows for an assessment of which policies and population attributes were most impactful in driving vaccination and ensuring efficient and equitable distribution among citizens. By focusing on the diverse strategies employed by different states in terms of (1) defining the entity responsible for distribution policy, (2) determining the groups eligible for vaccination, and (3) the timing for communication of distribution plans for vaccination, this descriptive investigation sheds light on the effectiveness of state-level interventions and contributes to a deeper understanding of how to manage large-scale public health initiatives. By identifying successful strategies and potential pitfalls, the study provides a roadmap for responding to future pandemics, ensuring that vaccination efforts can be swiftly and fairly implemented to protect public health.

## Introduction

The COVID-19 pandemic exposed significant vulnerabilities in global health systems and underscored the need for rapid and coordinated responses to combat emerging infectious diseases. The unprecedented scale and speed of the pandemic highlighted the importance of understanding the factors that contribute to successful vaccine distribution. Early studies highlighted the importance of effective policy strategies related to distribution—even above the efficacy of the vaccine itself—to achieve high rates of vaccination and reduce disease transmission [1]. Policy initiatives played a key role in managing vaccine supply and demand by setting guidelines for effectively distributing vaccines [2]. This study aims to identify those decisions and policy initiatives related to distribution during the early phases of the COVID-19 vaccination rollout that enabled rapid vaccination.

Investigating vaccine distribution is essential for understanding the impact of COVID-19 strategies, as well as for guiding future pandemic response strategies and improving preparedness for similar public health crises. Epidemiologists and public health experts alike agree that future pandemics are inevitable [3]. We argue that the COVID-19 pandemic presents a unique opportunity to study modern-day response strategies that are not confined to a specific geographic region or group of individuals, as might be seen by in more targeted outbreaks of other vaccine-preventable illnesses, like measles or dengue fever. Unlike other countries that managed responses at a national level, the United States handled the vaccination rollout at the state level, allowing us to examine the factors contributing to successful vaccination based on state and county-level differences. Some states were praised for their efficient vaccine distribution, while others were criticized for their lack of coordination [4]. By exploring each county’s responses to vaccination distribution in the United States daily during the vaccine rollout, our inquiry identifies the specific state-level distribution policies and county-level characteristics that impacted vaccine response.

Specifically, we consider the impact of (1) the entity responsible for distribution policy, (2) the determination and timing of groups eligible for vaccination, and (3) the timing of public communication related to distribution plans for vaccination. We also consider states’ political orientation. Further, we explore how factors that present barriers to vaccination at the local level—more specifically, the extent to which county residents are routinely under vaccinated, socioeconomic barriers impact citizens’ access to healthcare, health care systems are resource constrained, barriers to healthcare accessibility are present, and health care seeking can be defined as irregular among the population. We also consider the impact of race, as people of color carried the disproportional burden of the virus in terms of negative health outcomes and access to treatment, and more generally face longstanding disparities in access to healthcare [5].

In this inquiry, we define success as the percentage of eligible citizens who received at least one dose of a COVID-19 vaccine (henceforth referred to as “vaccinated”) in each county, measured daily throughout the initial U.S. vaccine rollout (i.e., for the first 6 months of 2021). This allows us to explore the differential impact of county characteristics and state-level policies on vaccination during the period during which supply of the vaccine exceeded demand. We argue that policies (e.g., eligible groups) were rendered less crucial when distribution polices were lifted and the vaccine was widely available to all citizens and easily accessible. The U.S. makes for an ideal context to explore policy differences using different counties as units of analysis, each acting as a “policy lab” in a natural context. This allows us to explore the different factors that contributed to vaccination, and to derive implications that can drive future emergency resource distribution strategies in the U.S. and beyond.

While other research has explored individual policy decisions as well as population characteristics that impacted the COVID-19 vaccine rollout, we know of no comprehensive inquiry that focuses specifically on vaccine distribution policy and seeks to highlight the differential impact of state-level policies while also exploring population characteristics at the county level. While extant work has considered the motivational factors that impact the decision to be vaccinated (e.g., competence, hesitancy), the World Health Organization also underscores the importance of understanding the “practical issues” of vaccine availability that are critical to ensuring that those who are motivated can be vaccinated [6]. Understanding the successes and challenges faced during the COVID-19 vaccine rollout can inform policies and practices for future pandemics, ensuring more efficient and equitable distribution of vaccines and other critical resources. The lessons learned from this pandemic can help build more resilient health systems and improve global preparedness for future health emergencies.

In the following sections, we review and discuss prior findings and identify how this study builds upon and extends extant research. Section 3 explains the data collection and methodology, and we report on our model and results in Sect. 4. We then explore how these results can guide policymakers in distributing vaccines or other healthcare resources in a pandemic or in the context of other emergencies where supply cannot keep pace with demand, rendering efficient distribution critical for individual and collective well-being. We conclude our research by discussing limitations and future research in Sect. 6.

## Literature review

Our work focuses on vaccine distribution strategy and the ways in which policies as well as barriers to vaccination impact citizens’ decisions to pursue the COVID-19 vaccine. We review previous literature on vaccination from two primary streams—that which considers the impact of distribution policy and population characteristics on vaccine outcomes. Below, we discuss each.

### Vaccine distribution policy – promoting vaccination

As scientists worked to develop effective vaccines to prevent the spread of COVID-19, researchers across disciplines—from management scientists to public health experts—highlighted the importance of creating effective implementation strategies to achieve high rates of vaccination [1]. This was supported by policy; the White House’s *Operation Warp Speed* aimed to accelerate both the development and distribution of the COVID-19 vaccine to ensure effective and equitable vaccine distribution [2, 7]. Prior work explores conceptual strategies for effective distribution [7]. Our study builds upon these, including work by Dai and Song that emphasizes the importance of research that leads to the development of effective distribution strategies [2]. We expand this conversation by empirically evaluating the different drivers on vaccination across U.S. counties.

While the task of vaccine production was underway, states began by identifying what government entity would manage statewide vaccine distribution efforts. Tasks assigned to these groups included defining which groups would receive priority, managing issues related to acquiring vaccine supplies, and updating guidelines for vaccination as new information became available [8]. Extant research that considers the development of COVID-19 vaccine policy in the U.S. finds that collaborative efforts and strategic decision making were key drivers to vaccination success [9–11]. We expand this line of research by considering the differential impact of government entities responsible for setting vaccine policy. Further, we employ a larger data set, both in terms of variables and number of observations, to empirically evaluate the effect of who should be responsible for vaccination rollout.

Designated responsible parties next decided what statewide plans would entail. Key to COVID-19 distribution polices were decisions related to eligibility. Consistent with the premise that the U.S. sought to mitigate the effects of the virus [10], vaccines were typically made available first to more vulnerable groups, like the elderly or those with underlying health conditions, as well as health care providers and first responders who were most likely to be in contact with others and, in turn, transmit the virus if infected [11]– [12]. In the context of vaccines, extant research suggests that the optimal distribution strategy for reducing illness and death due to vaccine-preventable illnesses may depend on vaccine efficacy. More specifically, when a vaccine is less effective, better public health outcomes are reached by distributing shots to high-risk populations; a more efficacious vaccine results in better outcomes when it is first distributed to those most likely to transmit the virus through contact with others [13]. Work by Cameron-Blake and colleagues presents a comprehensive review of COVID-19 vaccination policies across 185 countries, and observes that countries that aimed to eliminate the virus (e.g., New Zealand) prioritized border workers and economic sectors when defining eligible groups, while those aiming to mitigate the virus’s impact (e.g., the United States) tended to prioritize the elderly and healthcare workers [14]. Focusing on distribution, our work seeks to extend these findings to evaluate the ways in which different policies related to eligible group phasing impacted COVID-19 vaccination rates.

In addition to the plans themselves, states differed in terms of when they made their strategies for vaccination public. Exploring differences in communication timing is important as during the vaccine rollout—and particularly in the early stages of it—there was widespread confusion related to who was eligible; this was attributed to the county-level differences in distribution which left individuals unsure about the timing of their own eligibility [15]. Exploring when plans were made public contributes to the literature on policy communication to explore whether earlier communication impacted outcome measures—perhaps by providing clarity to mitigate confusion or allowing citizens to plan more effectively to be vaccinated.

Our research on vaccination success during the COVID-19 pandemic utilizes county-level data and daily vaccination rates, significantly expanding existing policy studies by providing granular insights into local vaccination efforts and outcomes. By analyzing localized data, we highlight the variability in vaccination success across different regions and identify key factors related to the who (responsible party), the what (eligible groups), and the when (communication timing) influencing these outcomes. The approach of looking at these simultaneously allows for a more nuanced understanding of how policies can be tailored to address specific community needs, ultimately contributing to more effective public health strategies.

### Population characteristics – barriers to vaccination

Public policies related to childhood vaccinations are known drivers of vaccination decisions [16], but there are also population-based factors that may drive outcomes. Generally, vaccinations for new or seasonal vaccines are correlated with standard vaccination rates [17], and studies show that vaccine hesitancy is an issue across the United States for all vaccines, including those for COVID-19 [18]. That said, there are communities with historically low vaccination rates (e.g., MMR, seasonal flu, COVID-19, etc.) relative to other areas. For example, rural communities are shown to have lower vaccination rates for COVID-19 as well as higher levels of overall vaccine hesitancy [19]– [20].

Extant literature shows that higher socioeconomic status (as defined by variables related to education, income, social class, and occupation) is associated with higher levels of vaccination for influenza [21]. This holds regardless of policies mandating vaccines; prior research shows no clear link between mandatory vaccination policies and higher immunization rates in most high-income countries [22]. This highlights the importance of considering cultural contexts and vaccine hesitancy when designing effective vaccination strategies. Other work examines the equitable allocation of COVID-19 vaccines in the United States, focusing on how different jurisdictions adopted measures to reduce inequities, including the use of disadvantage indices to prioritize vulnerable populations and the inclusion of vaccination sites to ensure equitable access [23].

Additionally, healthcare systems vary across the United States in terms of the extent of resources available for preventative care. In addition to geography, other barriers include limited financial resources and logistical issues, like lack of transportation. Following the logic that COVID-19 vaccination rates should track with patterns for other vaccines, these factors are critical to account for in terms of vaccine policy [17]. Our study considers these barriers to consider their role in vaccination outcomes.

Prior patterns of behavior may also drive vaccination behavior. Extant research on the drivers of vaccination demonstrates that routine care (e.g., preventative appointments) is associated with higher vaccination rates for children [24]. Further, in the context of childhood vaccinations, trusting relationships with healthcare providers are associated with higher vaccination rates [25] and extant literature demonstrates that provider support (e.g., reminders and discussions at appointments) can result in increased adult vaccinations [26].

Our research underscores the importance of data-driven frameworks in managing public health crises. We significantly expand demographic and vaccine hesitancy studies by providing detailed insights into how various population characteristics influence vaccination outcomes. This granular approach allows for the identification of specific demographic groups that may face unique challenges, thereby informing targeted interventions to reduce vaccine hesitancy and improve equitable access. The key contribution of our study is combining the population characteristics described in this section with the policy characteristics described in Sect. 2.1. This allows us to explore relationships among the variables, significantly expanding previous research. Further, our research is the first—to our knowledge—with such a robust dataset, allowing us to build on theoretical research in vaccine distribution while yielding deeper insights that can drive better public health outcomes. We describe our data next.

## Data collection and methodology

In this work, we capture data from various disjoint sources to examine specific measures that U.S. states took to effectively distribute the limited supply of the vaccine, as assessed by the percentage of the population in each county each day that received at least one dose of the vaccine. Receiving a first dose means that an individual has access and has chosen to receive the vaccine, and during the time period studied most citizens who received a first vaccine went on to complete the two-dose series [27]. We also consider data describing the vulnerabilities of populations from localities within U.S. states (i.e., county-level) and include control variables like population density and the political party holding elected office. Our data is heterogeneous and scraped from multiple publicly available sources (for a list of the primary variables and their sources, see Table 1). Data was collected every day from January 1 to June 31, 2021, for 2,876 U.S. counties (and county equivalents) for the percent of population vaccinated and vaccine eligible groups based on age and occupation.^1^All other variables were time invariant across the period explored. The January through June time frame was selected because demand for the vaccine exceed supply; after this time period state eligibility requirements were lifted, rendering established distribution polices outdated as anyone could access the vaccine. In the sub-sections that follow, we define and motivate the key variables collected. A summary of the variables collected, including data sources, can be found in Table 1.

**Table 1:** Variable name, source, and coding

Variable	Coding	Time Step	Geographic Resolution
Share of Population Administered at least one dose [28]	*Continuous*: 0 to 100	Daily	County
Vaccine Responsibility [29]	*Categorical*: 1 = committee or task force 2 = state department of public health 3 = other (multiple groups involved)	Constant	State
CVAC [30]	*Categorical*: 0 = below-median CVAC score 1 = above-median CVAC score	Constant	County
Eligible Group Phasing [29]: (1) Share of county population eligible via age-based categories (2) Number of (occupation) targeted groups eligible	*Continuous*: (1) Sum of binary indicator = 1 if a given age category was eligible on that date, multiplied by the share of the county in that age category (2) Sum of binary indicators = 1 if an occupational or other targeted group was eligible on that date	Daily	(1) County (2) State
Date Plan Communicated to Public [29]	*Continuous*: Actual date state vaccine rollout plan was communicated to the public	Constant	State
Population Density [31]	*Continuous*: (Standardized) population per square mile; ranges from − 0.15 to 40 standard deviations	Constant	County
Demographic Composition [30]	*Continuous*: Percent of population (0 to 1) of each the following racial and ethnic groups: • White, non Hispanic • Hispanic • American Indian • Asian • Black • Native Hawaiian	Constant	County
State Political Orientation [32]	*Categorical*: 1 = Republican 2 = Democratic 3 = Split legislative and governor control	Constant	State

### Distribution policy factors

#### Vaccine responsibility

Vaccine responsibility identifies the government entity responsible for vaccine distribution in each state. In considering this variable, we seek to shed light on the efficacy of various structures for emergency preparedness in the domain of public health. States relied upon either an ad-hoc COVID-19 committee, an established department of public health, or in some instances planning involved more than one entity. We created a categorical variable to assess the impact of vaccine responsibility (see Table 1). A summary of state approaches to the responsible public health organization can be found in Table 2.

**Table 2:** Distribution of responsible entity

Responsible Entity	Percent of Dataset
Specialized Committee/Task Force	48%
Department of Health	30%
Other (multiple groups involved)	22%

#### Eligible group phasing

Guided by the ACIP, each state and county determined which citizens would be eligible for the COVID-19 vaccine and when during the time when demand for the vaccine exceeded supply. In our inquiry, we seek to understand whether broadening the recommended ACIP phases (i.e., including more eligible citizens than suggested by the guidelines) led to increased rates of vaccination, or whether those states that aligned with recommendations were able to roll out their vaccination plans more methodically and efficiently, resulting in more vaccinated citizens. The latter would suggest that distribution plans left to more localized government entities (e.g., states), versus a federal government, might result in the more efficient allocation of scarce resources during an emergency. We considered both targeted groups and age-based criteria for eligibility in our model.

We first collected data on the day each targeted group (identified by the ACIP guidelines) was eligible for the vaccine in each state. Targeted groups were based on occupation or high-risk medical conditions:

- first responders
- grocery store staff
- high-risk medical conditions
- K-12 educators
- frontline essential workers
- food processing workers
- postal workers

**Fig. 1:**
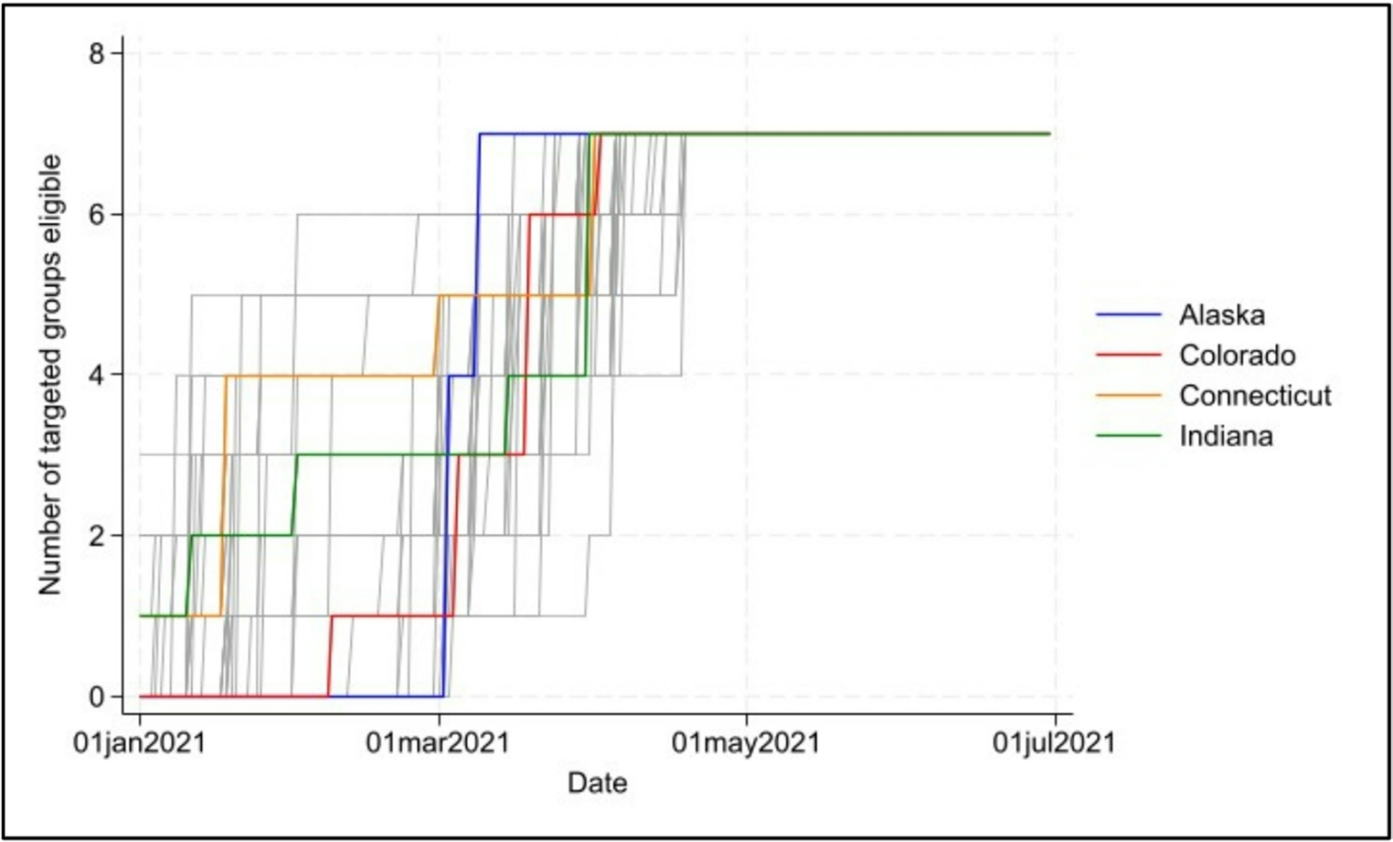
Eligibility count of eligible groups across vaccine rollout

For consideration of eligibility for targeted groups, we summed up the total number of these groups eligible to receive the vaccine each day. Thus, for every observation (i.e., for each county on each day) in our data set, we had a variable ranging from 0 (none of these were eligible) to 7 (all listed groups were eligible). Larger scores indicate states with more liberal policies toward opening vaccine eligibility, while smaller scores indicate a stronger adherence to those guidelines suggested by the federal government (see Table 1). Figure 1 shows the number of targeted groups eligible over the time of the initial vaccine rollout. Four states are highlighted to show differences: Alaska was early to open eligibility to more groups; Connecticut opens later in the timeframe; Colorado has consistent eligibility; Indiana falls between these.

**Fig. 2:**
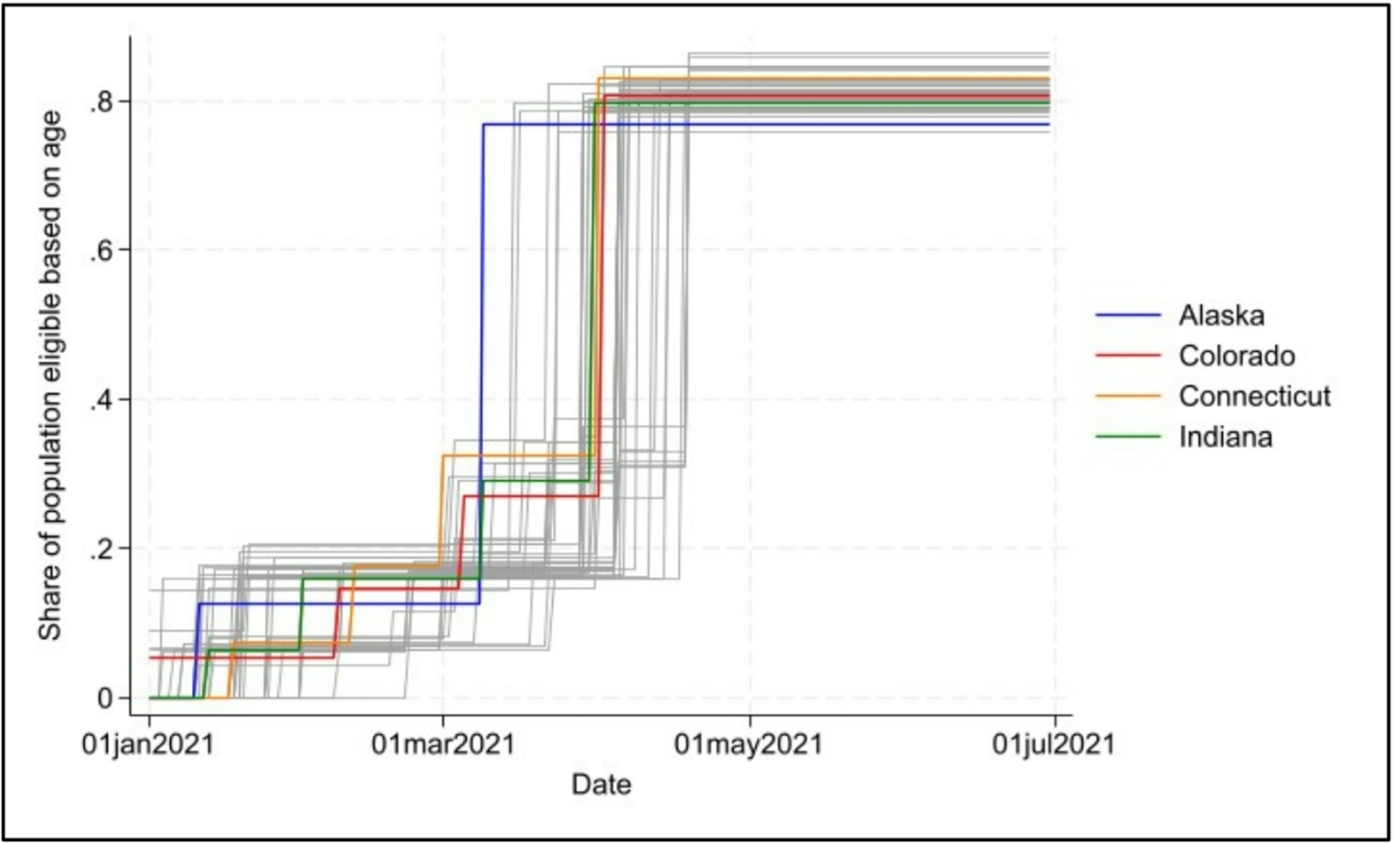
Share of population eligible based on age across vaccine rollout

Additionally, we created a variable indicating the share of the population eligible based on age. This was calculated considering age restrictions (e.g., 55 and over) and the share of the county population at or above the age specified in the eligibility guidance, giving an overall assessment of the number of people without critical community roles (e.g., first responders) or high-risk medical conditions who were able to receive the vaccine. This variable tracks how quickly states were able to lift all restrictions on vaccine eligibility, and an overview can be seen in Fig. 2, which demonstrates the variation on the lifting of restrictions across states. Alaska was the first state to lift restrictions and open vaccine eligibility to all residents over the age of 16 on March 9, 2021.

#### Plan communication

Additionally, we collected data to reflect the date each state initially communicated their plan to state residents. Most states (62%) communicated their plan to the public on October 16, 2020, when the draft distribution plan was due to the CDC. As discussed in the preceding section, state-level differences in distribution resulted in a sense of uncertainty related to who was eligible for vaccines at various points in time throughout the vaccine rollout [15]. We consider when the plans were made public to determine if earlier communication impacted outcome measures—perhaps by providing clarity to mitigate confusion.

### Population vulnerability

**Fig. 3:**
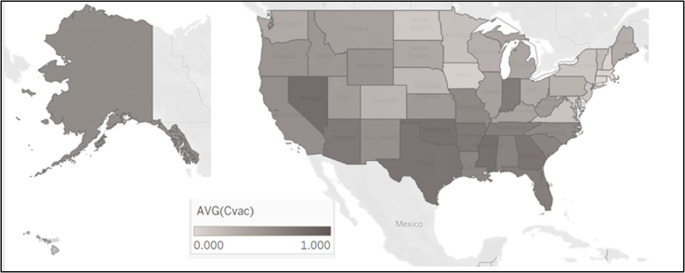
Average CVAC of each State

To account for population-based vulnerabilities likely to impact an individual’s decision to receive the vaccine, we collected the COVID-19 Vaccine Coverage Index (CVAC) for each county across our set of U.S. states. The CVAC is designed to capture barriers to vaccination. For more information on the scale including sources and validation metrics, we refer the reader to Surgo Ventures [33]. The five sub-scales that comprise the CVAC index for each county capture the extent of (1) historic under vaccination, (2) socioeconomic barriers that impact access to healthcare, (3) resource-constrained health systems, (4) barriers to healthcare accessibility, and (5) irregular care-seeking behaviors. Scores on these measures range from 0 (least concerning) to 1 (most concerning) and are averaged to form an overall measure for each county. We include a dichotomous variable of 1 (0) if the county’s CVAC is above (below) the average across all counties in the dataset to account for community factors that may impact the access to and utilization of vaccines that provide pandemic relief. Figure 3 shows the average CVAC score by state, with the darker shading demonstrating relatively stronger barriers to vaccination based on the defined CVAC factors.

### State and local control measures

#### Political control

Extant research underscores the politicization of responses to the COVID-19 pandemic. Inquiries into the differences in state political affiliation demonstrate differences in terms of a variety of outcome measures. For instance, Democrat-led states tended to have stricter public health measures, resulting in slower growth in infection rates [34]. While Republican-led states had lower rates of incidence and death early in the pandemic, by the summer of 2020 this pattern reversed [35]. As the views and policies of elected officials are likely to align with many state citizens, we consider differences in the political orientation of the state. Evidence shows that counties with a high percentage of Republican voters have lower vaccination rates [35]; in line with this, we explore whether party leadership impacts vaccination.

**Fig. 4:**
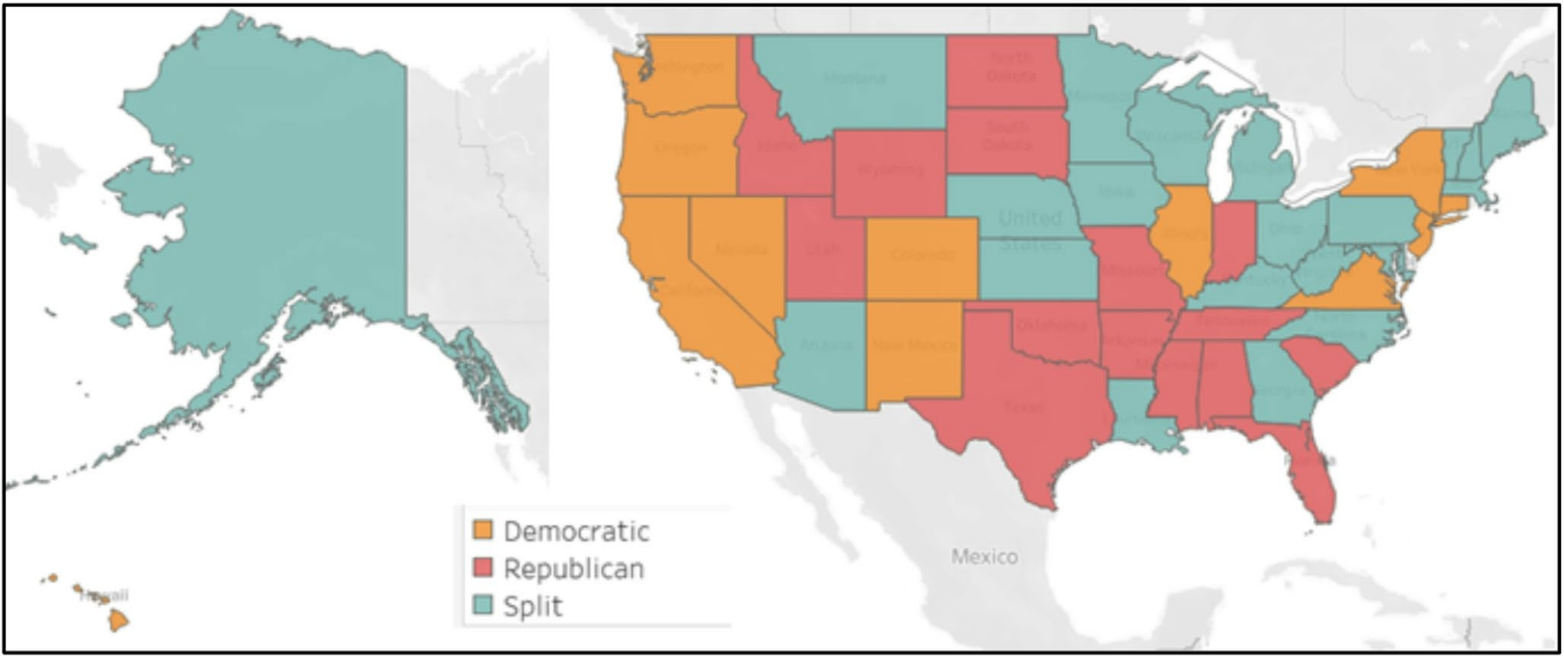
Party affiliation by state

We define party affiliation as a function of the governor and the state legislative control. State control is labeled as “split” if the legislative control and governor are affiliated with different political parties. State control is characterized as Democrat or Republican when the governor and legislature are of the same party. The makeup of U.S. states in terms of party affiliation is displayed in Fig. 4.

#### Population density

We recognize that the ease of healthcare distribution may be impacted by the population density of each county and thus include this as a control variable in our model. It may be the case that more (vs. less) densely populated counties experience difficulties in distribution due to aspects of the supply chain (e.g., shipping volume) as well as logistics in administration (e.g., catering to different populations or complexities based on a larger volume of citizens seeking care). On the other hand, it is possible that more densely populated counties were able to more efficiently vaccinate their population because of ease of access and fewer complexities associated with managing the supply chain and distribution of vaccines. To assess the impact, we consider a standardized measurement of population per square mile for each county. As expected, the distribution of population per square mile by counties is highly skewed, hence a standardized measurement is more meaningful.

#### Demographics

The CDC identified vaccine equity as an important goal during distribution [36], recognizing that underrepresented groups have historically faced longstanding disparities in access to healthcare would likely carry the disproportional burden of the COVID-19 virus [5]. To explore how the race-related demographics of a county impact vaccination rates, we consider the percent of the following groups in our model: Hispanic, American Indian/Alaska, Asian, Black, Native Hawaiian/Pacific Islander, and White.

Table 1 provides a summary of the variables included in the model, the disparate sources of our data, and details on variable specification. In the next section, we describe our methodology and results related to understanding the impact of our identified policy factors, population vulnerability factors resulting in barriers to vaccination, and state and local community characteristics on vaccination outcomes.

## Model development and results

**Fig. 5:**
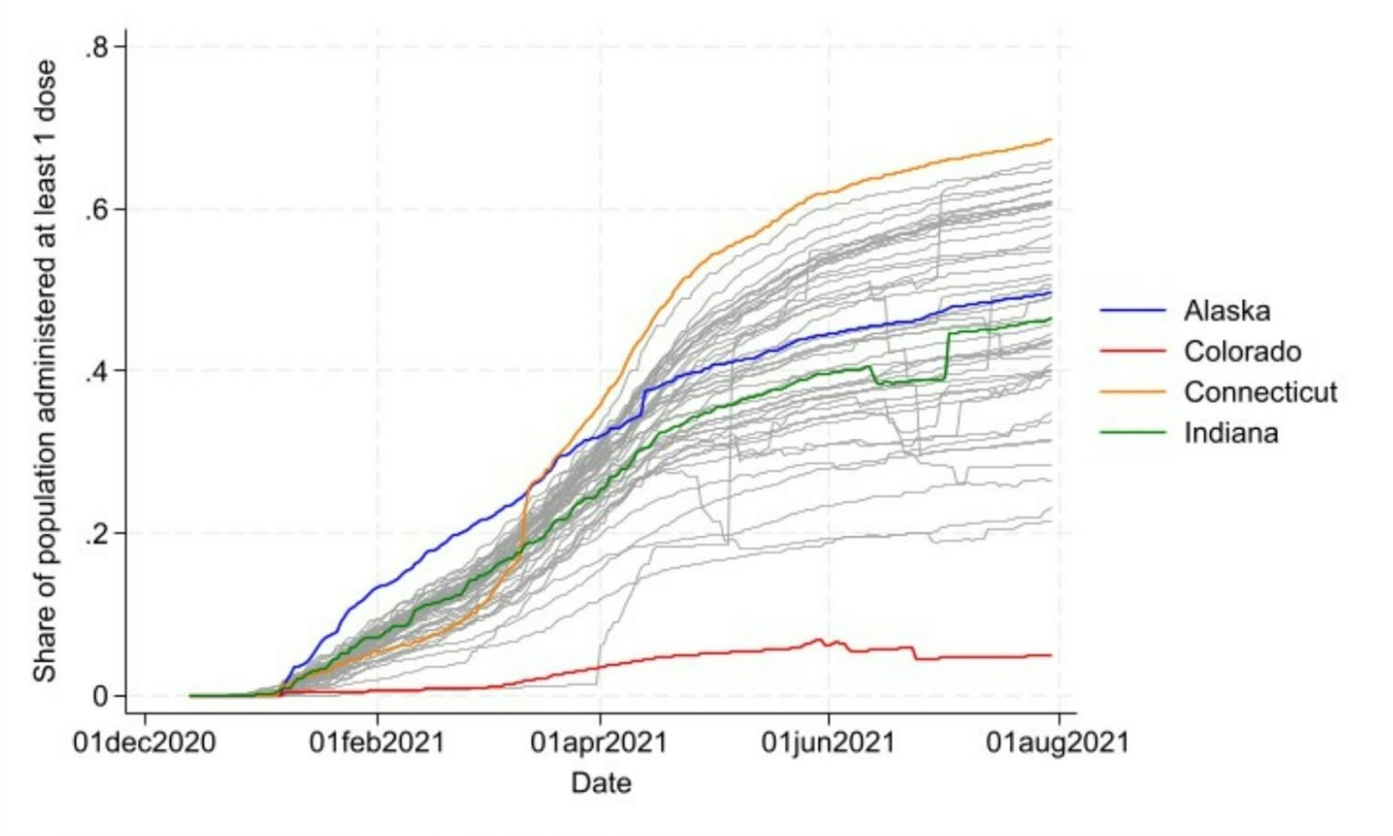
Vaccination rates for state over time

In the analyses described next, we explore how state-level policies regarding vaccine distribution affect pandemic response—as measured by the county’s percent of eligible population vaccinated with the first dose—when accounting for county-level factors. The timeframe that we focus on is the initial rollout, or when demand for the vaccine exceeded supply. This is a critical time frame to explore as it was during this time when policy not merely demand but dictated access. Demand exceeding supply in the early months of vaccine rollout is supported by various policy sources citing a supply-demand imbalance [36, 37], as well as patterns in our dataset. The percent of population vaccinated was collected daily for every county in the U.S. from January 2021 through June 2021 from the CDC [28]. A summary of vaccination rates throughout the vaccine rollout and identification of the states that were top and bottom performers based on these rates are shown in Table 3; Fig. 5.

**Table 3:** Summary of vaccination rates and top/bottom performers by state

Date	Average Vaccination Rate	Top Vaccination Rate	Bottom Vaccination Rate
January 1, 2021	0.07%	0.9% (Florida)	0% (41 states)
February 1, 2021	6.59%	13.4% (Alaska)	0.37% (New Hampshire)
March 1, 2021	13.11%	21.7% (Alaska)	0.85% (New Hampshire)
April 1, 2021	25.9%	35.9% (Connecticut)	3.4% (Colorado)
May 1, 2021	36.8%	54.4% (Connecticut)	5.2% (Colorado)
June 1, 2021	42.6%	62.1% (Connecticut)	6.3% (Colorado)

Our model explores daily variations in the percent of population vaccinated between counties explained by state- and policy-level factors (some that do not change over time and others, such as phased eligibility, that do) while controlling for daily fixed effects to account for any nonlinear national time trends in vaccination. That is, we seek to explain what factors played a role in some counties vaccinating more or less than the average county on that day. To build our model, a balanced panel of county and county equivalents were observed every day during our inquiry timeframe.

Our panel regression fixed-effects model has 520,556 observations and an R-square of 0.648. This analysis allows us to treat each county on each day as a unit of observation to provide insight as to what policy (i.e., responsible entity, vaccine eligibility, plan communication date) and population variables (i.e., CVAC) impact the percentage of citizens vaccinated at each point in time when controlling for important area characteristics (i.e., political orientation, population density, demographic composition).

It is reasonable to assume all explanatory variables are independent, as all correlation values are reasonably close to zero; most values fall between − 0.1 and 0.1, indicating a very weak (or essentially no) relationship between variables (see Appendix Table 7). We do explore a mild relationship between the responsible entity and CVAC (i.e., correlation value of 0.1117; see Appendix Table 7) by including an interaction term. There is one high correlation—a value of 0.8787— between age-eligible groups and occupation-eligible groups. This is a function of time, such that as time passes, both variables increase as more people become eligible in each state (as determined by targeted groups or age). This is not concerning since our model includes a day fixed effect. No evidence of multicollinearity is seen in our model.

Counties are balanced (weighted) so that states with a large (or small) number of counties are not over (or under) represented in the model. Non-linear time trends are accounted for with the day fixed effect. The model to predict the share of population vaccinated for each *t* day in each *i* county includes contextually relevant variables plus our interaction term:

$$\begin{array}{c}Percent\;Vaccinated_{ist}\\=\beta_0+\beta_1\;Responsible\;Entity_s\\+\beta_2CVAC_i+\delta_1Responsible\;Entity_s\ast CVAC_i\\+\beta_3Percent\;Age\;Eligible_{ist}\\+\beta_4\;Targeted\;Groups_{st}\\+\beta_5Date\;Plan\;Communicated_s\\+\beta_6\;Population\;Density_{is}\\+\beta_7\;Demographic\;Composition_{is}\\+\beta_8\;Political\;Orientation_s+\gamma_t+\varepsilon\\\end{array}$$

For county *i* in state *s* on date are $t.\gamma_t$ day fixed effects, and standard errors are heteroskedasticity-robust. No evidence of multi-collinearity was found.

This model was considered over three time periods. The full model uses data from January 1, 2021 to June 31, 2021. Then, to identify factors determiningg initial versus later success, two additional models using a subset of the data were created: a model over the period of January 1, 2021 to March 31, 2021 and another model over the period April 1, 2021 to June 31, 2021. The results of these multivariate regression models can be seen in Table 4.

**Table 4:** Comparison of model results across vaccine rollout

Policy and Population Factors	Time Frame						
	Jan – June			Jan – Mar		Apr – June	
**Responsible Entity** Specialized Task Force Department of Public Health	2.622*** 0.968***	(0.100) (0.098)		2.419*** 1.718***	(0.081) (0.082)	2.785*** 0.340**	(0.170) (0.166)
**CVAC**	0.913***	(0.079)		2.751***	(0.067)	−0.857***	(0.134)
Responsible Entity*CVAC Specialized Task Force|High CVAC Department of Public Health|High CVAC	−2.266*** −1.074***	(0.108) (0.122)		−2.236*** −1.718***	(0.087) (0.092)	−2.235*** −1.05***	(0.118) (0.211)
**Eligible Groups** Age-based Groups Targeted Groups	7.814*** −0.505***	(0.188) (0.013)		2.570*** −0.107***	(0.207) (0.011)	20.26*** −3.868***	(0.492) (0.136)
**Date Plan Communicated**	−0.170***	(0.001)		−0.077***	(0.001)	−0.252***	(0.002)
**Population Density**	0.131***		(0.017)	0.062***	(0.007)	0.173***	(0.023)
**County Demographic Composition** Hispanic American Indian/Alaska Native Asian Black Native Hawaiian/Pacific Islander	−9.632*** 17.88*** 83.48*** −4.281*** −212.3***	(0.309) (0.234) (1.045) (0.113) (10.61)		−7.889*** 17.06*** 20.73*** −3.508*** −43.04***	(0.184) (0.275) (0.562) (0.075) (7.841)	−10.42*** 20.59*** 145.5*** −4.697*** −338.8***	(0.582) (0.374) (1.878) (0.192) (19.79)
**State Political Orientation** Republican Split Control	−1.263*** 0.927***	(0.082) (0.791)		0.322*** 1.061***	(0.054) (0.049)	−2.376*** 0.935***	(0.145) (0.139)
**Constant**	3,778***	(29.420)		1,703***	(20.180)	5,631***	(51.260)
**Observations**	520,556			258,840		261,716	
**R-squared**	0.648			0.671		0.278	
**Fixed Effects**	Day			Day		Day	

*** *p* < 0.01, ** *p* < 0.05, * *p* < 0.1; robust standard errors in parentheses

## Discussion of factors driving vaccination

Next, we discuss the factors that contributed to the success of vaccine distribution, as determined by our regression model. A high-level overview of our data-driven observations and actionable insights are shown in Table 5. We discuss these in more detail, identifying factors that played a role across the timeframe of the vaccine rollout.

**Table 5:** Data-driven observations and associated insights

Variable	Data-Driven Observation	Future Action
Vaccine Responsibility + CVAC	States that set up a COVID-19 committee/task force were most effective in driving vaccination. The advantages of setting up a specialized task force are immediate in high CVAC counties. There is a strong long-term advantage for counties with a low CVAC to set up a Specialized Task force	Establish a dedicated, cross-functional vaccine distribution team early—especially in resource-limited settings. Include diverse perspectives (e.g., public health, government, community leaders) to improve planning and trust.
Eligible Group Phasing	The number of targeted groups for which vaccines were made reduced vaccination rates.	Relaxing restrictions early may lead to desired changes in overall vaccination.
	Opening eligibility based on age had a positive impact on overall vaccination.	Prioritize simple, broad eligibility criteria (e.g., age-based) to accelerate rollout. Avoid overly complex or fragmented group targeting in early phases.
Plan Communication	Earlier communication positively impacted monthly vaccination rates.	Communicate distribution plans clearly and early to build public confidence and support informed decision-making.
Political Party Affiliation	Split political control (i.e., representation from Republican and Democratic leaders) led to higher rates of vaccination.	Encourage bipartisan or multi-stakeholder collaboration in public health planning to ensure balanced, inclusive policies.
Population Density	Population density positively impacted vaccination.	Tailor distribution strategies to geographic context. In rural or low-density areas, invest in mobile clinics, outreach, and transportation support to improve access.

### CVAC & responsible entity

Recall that our model included the CVAC measurement as 1 or 0, corresponding to whether the county’s CVAC was above (1) or below (0) the CVAC mean of 0.58; raw CVAC score ranges from 0 (least concerning) to 1 (most concerning). Because the CVAC reflects known barriers to vaccination, the expectation is that as the CVAC measurement increases, vaccination rate decreases. Our model shows this overall pattern.

Additionally, and as discussed in the previous section, we also consider the interaction of CVAC with our categorical variable indicating the responsible entity for COVID vaccines in that state. Therefore, coefficients should be interpreted with this interaction in mind. The omitted category for responsible entity is “other”– that is, multiple groups were involved in the decision. Our full model shows that a specialized task force yields the highest vaccination rates for both low and high CVAC measurements. This is also true in both the early (January-March) and later (April-June) phases of the vaccination rollout. Low CVAC measurements (i.e., those characterized as least concerning; shown in the Table 6, in gray) and a specialized task force is associated with the greatest increase in population vaccinated—even higher than all groups with high CVAC measurements in the full model.

Over time, a specialized task force has the highest positive effect on vaccination rates for low CVAC counties, as seen in the January to March and April to June models—with slightly higher rates seen in the later model. This is also true for high CVAC counties, but the impact reverses over time. When considering the initial success of vaccination (January to March), the highest vaccination rate is seen with specialized task force and high CVAC measurements. However later (April to June), high CVAC counties have negative vaccination rates regardless of the responsible entity, but specialized task force remains the best entity (i.e., is associated with the smallest negative rate). This implies that the advantages of setting up a specialized task force are immediate in high CVAC counties, an effect which lessens over time; a more consistent long-term impact is seen in low CVAC counties. For low CVAC counties, the lowest vaccination rate occurs when multiple entities are involved in the vaccination rollout. This supports the idea that involving too many decision makers may hinger efficient progress. It is interesting that in the last three months of our explored timeframe, vaccination rates fall for all combinations except low CVAC with specialized task force. Hence, there is a strong long-term advantage for counties with a low CVAC to set up this type of entity.

Our findings point to the importance of the party responsible for distribution and administration, demonstrating that those states that assigned responsibility to a dedicated task force observed higher levels of vaccination. Interestingly, these task forces were not just comprised of policy leaders, but also representatives from vulnerable populations, health insurers, pharmacists, educators, and healthcare providers. In future disaster situations involving limited resources, states and other government agencies should consider task forces comprised of various community members.

**Table 6:** Model coefficients for interaction terms

Time Frame	Jan – June	Jan - Mar	Apr - June
Specialized Task Force & High CVAC	1.269	2.934	−0.307
Specialized Task Force & Low CVAC	2.622	2.419	2.785
Department of Public Health & High CVAC	0.807	2.751	−1.567
Department of Public Health & Low CVAC	0.968	1.718	0.340
Other & High CVAC	0.913	2.751	−0.857
Other & Low CVAC	0.000	0.000	0.000

### Population eligibility

Both the share of the population eligible to receive the vaccine and the number of targeted groups eligible to receive the vaccine were significant in our model. Unsurprisingly, the more people in the county that are eligible for the vaccine by age, the higher the vaccination rate. Of more importance, we find that adding more targeted eligible groups sooner does not help to improve vaccination rates; in fact, as more targeted groups are added (beyond age groups) the vaccination rates are lowered. It seems as though adding eligibility groups too quickly hinders vaccinations.

Contrary to this finding, age-based eligibility helped vaccination rates. This is likely because age-based qualifications were clear and easily understood. Other eligibility groups established by the ACIP were perhaps vaguer (e.g., healthcare workers), therefore blurring the lines of who meets the criteria for receiving the vaccine. This is an important observation; while eligibility groups were designed to control the limited supplies of the vaccine during the initial rollout and to ensure that vulnerable groups and those with increased exposure were vaccinated first, many U.S. citizens were confused about eligibility, perhaps also due to differences across states [15]. In the context of vaccines, extant research suggests that the optimal distribution strategy for reducing illness and death due to vaccine-preventable illnesses may depend on vaccine efficacy. This finding is consistent with prior work that supports better vaccination outcomes when shots are given first to high-risk populations, rather than those more likely to transmit a virus, such as essential workers [13].

It should also be noted that the relationships between eligibility groups became more pronounced later in the rollout. In the first three months of vaccine rollout, the share of the population eligible by age was significant, with a coefficient of 2.57. However, lifting age restrictions became more important in April through June with a coefficient of 20.26. That is, as 10% of the share of the population eligible based on age increased, we noted a 2.26% increase in vaccination rates. As more groups became eligible for the vaccine, we saw the opposite effect: more groups actually lowered vaccination rates to a greater degree in April through June.

### Government control and communication

Our model demonstrates evidence that providing information on vaccination plans sooner improved vaccination rates. This supports the idea that plans aimed at effective communication and planning may have been more successful in promoting vaccination outcomes. This, along with our findings related to eligibility in the preceding section, suggests that policymakers would be wise to focus on speed rather than methodology when resources are constrained in future scenarios. Interestingly, this contradicts calls from the medical community to consider the unique characteristics and experiences that differentiate communities and to plan vaccine distribution around them [38].

As well, state political control contributed to the vaccine distribution rate. Interestingly, those states that were split—having Democratic and Republican control (i.e., through legislature and governor)—saw higher vaccination rates then states that were exclusively Democratic or Republican. This is a significant finding in that it shows that different parties working together were stronger than a single party control in terms of vaccination outcomes. States whose political orientation was Republican saw lower vaccination rates than Democrat controlled states in the full model, which explored vaccination from January to June. However, in the short-term model that explored vaccinations in January through March, Republican controlled states had higher vaccination rates then Democratic states; this was still under the rate observed when political control was split.

### County demographics

In addition to exploring *how many* people were vaccinated, we also explore how the demographic make-up of a county shaped the overall vaccination rate. Our model shows there is a slight but significant positive effect on vaccination rates for more densely population counties. Additionally, our results reveal that jointly, race does influence vaccination rates. However, all races considered in the model are significantly different from the baseline of the White, non-Hispanic citizen group. Counties with higher shares of American Indian/Alaskan Native and Asian races had better vaccination rates than those with more White, non-Hispanic populations; counties with higher Hispanic, Black, and Hawaiian/Pacific Islander shares had lower vaccination rates than those with more White, non-Hispanic populations. It is also worth noting that the positive significance of the vaccination rate of Asians and the negative significance of Pacific Islanders became much stronger in the second half of the vaccination period (April-June).

## Conclusion

In this inquiry, we explore the impact of different distribution policy decisions that work alongside factors that relate to population vulnerability on the success of healthcare outcomes, using the dissemination of the COVID-19 vaccine as a case study. While experts and studies have looked at some of the variables in our analysis separately, we know of no such study that considers a comprehensive list of factors specifically related to distribution policy in the COVID-19 vaccination context. Moreover, we utilize a fixed effects multivariate regression model that allows us to describe which factors were significant across the vaccine rollout, and which were less impactful in driving a pandemic response plan. The key findings from our analysis are:

- Dedicated task forces boosted COVID-19 vaccination rates.
- More liberal age-based eligibility policies were more effective than opening eligibility to more targeted groups at increasing COVID-19 vaccination rates.
- Early communication enhanced COVID-19 vaccination.
- Split political control (i.e., Democratic and Republican leadership) led to better COVID-19 vaccination outcomes.
- Demographics and population density influenced vaccination outcomes.

This research is not without limitations. While we focus on factors related to distribution policy, we also recognize that there are additional variables that are not captured in our model that might be considered as drivers of healthcare-related behaviors. As such, this work should be considered within the broader literature that considers additional factors and different health-related outcomes (e.g., COVID-19 infections).

It is also important to note that our research design is not causal in nature. There likely are other factors determining vaccination rates that are correlated with our regressors—including, for example, mask mandates and lockdown enforcement. Much of this variation should be captured in the vaccine hesitancy (CVAC) measure and state political control, but our results should be primarily read as observational, and addressing the question of *what policy and population factors are associated with a county’s successful vaccination rollout?*

While the insights here offer a retrospective view of the COVID-19 vaccine distribution, our findings might also aid in the distribution of future pandemic relief resources. It is our hope that insights like those presented here might offer municipalities, states, and countries a guide as to what policy and population factors might be considered in their pandemic response efforts. Indeed, decision makers would be wise to use the lessons learned from the prior pandemic in drafting and evaluating plans for other infectious diseases, and particularly those for which preventative measures like vaccination are available in smaller quantities than they are demanded [39]. This may also hold true for other scarce resources in disaster situations; resources including housing, water, and necessary household products might be in limited supply, with states needing to quickly implement plans to distribute them.

## Data Availability

The data used in this study were obtained from publicly available sources. All relevant sources and links to the datasets are cited within the manuscript.

## Appendix

**Table 7:** Correlations between policy and population variables

	Responsible Entity	Eligible Groups (Age)	Eligible Groups (Targeted Groups)	Date Plan Communicated	State Political Orientation	CVAC
**Responsible Entity**	1					
**Eligible Groups (Age)**	−0.0175	1				
**Eligible Groups** **(Targeted Groups)**	−0.0294	0.8787	1			
**Date Plan Communicated**	0.0950	0.0014	−0.0506	1		
**State Political Orientation**	−0.0883	0.0348	−0.0362	−0.0016	1	
**CVAC**	−0.1117	0.0391	−0.0201	0.0569	0.0070	1

## Footnotes

1 All states except Texas and Hawaii were included in our dataset (daily data for these states were unreported).
